# Functional and ecological diversification of underground organs in *Solanum*

**DOI:** 10.3389/fgene.2023.1231413

**Published:** 2023-10-10

**Authors:** Edeline Gagnon, Ludwig Baldaszti, Peter Moonlight, Sandra Knapp, Caroline E. R. Lehmann, Tiina Särkinen

**Affiliations:** ^1^ Department of Integrative Biology, University of Guelph, Guelph, ON, Canada; ^2^ Tropical Diversity Section, Royal Botanic Garden Edinburgh, Edinburgh, United Kingdom; ^3^ Chair of Phytopathology, TUM School of Life Sciences, Technical University of Munich, Freising, Germany; ^4^ School of GeoSciences, University of Edinburgh, Edinburgh, United Kingdom; ^5^ Botany, School of Natural Sciences, Trinity College Dublin, Dublin, Ireland; ^6^ Natural History Museum, London, United Kingdom

**Keywords:** rhizomes, tubers, root sprouters, geophytes, range size, niche breadth, niche shifts

## Abstract

The evolution of geophytes in response to different environmental stressors is poorly understood largely due to the great morphological variation in underground plant organs, which includes species with rhizomatous structures or underground storage organs (USOs). Here we compare the evolution and ecological niche patterns of different geophytic organs in *Solanum* L., classified based on a functional definition and using a clade-based approach with an expert-verified specimen occurrence dataset. Results from PERMANOVA and Phylogenetic ANOVAs indicate that geophytic species occupy drier areas, with rhizomatous species found in the hottest areas whereas species with USOs are restricted to cooler areas in the montane tropics. In addition, rhizomatous species appear to be adapted to fire-driven disturbance, in contrast to species with USOs that appear to be adapted to prolonged climatic disturbance such as unfavorable growing conditions due to drought and cold. We also show that the evolution of rhizome-like structures leads to changes in the relationship between range size and niche breadth. Ancestral state reconstruction shows that in *Solanum* rhizomatous species are evolutionarily more labile compared to species with USOs. Our results suggest that underground organs enable plants to shift their niches towards distinct extreme environmental conditions and have different evolutionary constraints.

## 1 Introduction

Roots and underground storage organs are central to the ability of a plant to tolerate stress and disturbance and persist and compete across a diversity of environments (Bond and Midgley, 2001; Linder et al., 2018; Archibald et al., 2019; Ottaviani et al., 2020). Evolution of plant lineages to different environments have thus far shown the importance of aboveground traits such as growth form, leaf traits, photosynthetic pathway, and hydraulics (Edwards and Smith, 2010; Pittermann et al., 2012; Schmerler et al., 2012; Ogburn and Edwards, 2015; Zanne et al., 2015; 2018; Gagnon et al., 2019), but we still have a poor understanding of belowground traits, and how they relate to aboveground plant traits and ecosystem function (Weigelt et al., 2021). While assessing belowground organs is daunting (Klimešová et al., 2019; Tribble et al., 2021b; Freschet et al., 2021), it is needed for a holistic understanding of plant evolution and adaptation to diverse environments.

The placement of re-sprouting organs (i.e., buds) belowground in plants known as geophytes (Raunkiaer, 1934) is a major plant architectural trait related to the development of underground organs. The placement of buds belowground enables geophytic plants to survive and prosper in harsh environments (Ott et al., 2019), resprouting post-dormancy enabling avoidance of temperature and rainfall seasonality extremes (e.g., drought and heat stress, frost), as well as disturbances related to fire and grazing (e.g., geoxyles in African savannas; Maurin et al., 2014; Meller et al., 2022). Previous studies have noted belowground bud banks in environments with frequent disturbance (Fidelis et al., 2014; Pausas et al., 2018) and also in arid climates (Rundel, 1996; Parsons and Hopper, 2003; Procheş et al., 2006; Sosa and Loera, 2017). Geophytic plants are also common in temperate climates in both woodlands (Whigham, 2004) and grasslands (Herben and Klimešová, 2020), as well as in montane environments and arctic regions (Klimešová et al., 2011; Klimešová et al., 2012).

Beyond belowground bud placement there is wide variation in the morphology of underground organs in geophytic plants. Some geophyte species produce underground lateral stems termed rhizomes, that enable clonal reproduction and lateral spread enabling underground dominance in competitive environments, such as tropical grasslands (Klimešová et al., 2018; Ottaviani et al., 2020). This ability can also enable them to escape from repeated fire and herbivory (Klimešová et al., 2021; Meller et al., 2022). Species with adventitious buds on roots are functionally similar to rhizomatous species as they enable rapid resprouting from underground stems (Ott et al., 2019). The competitive advantage of rhizomes is apparent in the list of world’s most invasive plant species of which many are rhizomatous (e.g., see Japanese knotweed (*Reynoutria japonica* Houtt.), Kahili ginger (*Hedychium gardnerianum* Sheph. ex Ker Gawl.), and cogongrass (*Imperata cylindrica* (L.) Raeusch.); https://web.archive.org/web/20160304230622/http://www.iucngisd.org:80/gisd/100_worst.php). In addition, the ability to disperse laterally underground can allow plants to compensate for lower nutrient availability by increasing their spatial extent (February et al., 2019). Soil properties could also play a considerable influence on the capacity of plants for lateral dispersal, with the expectation that less compact soils, with higher contents of sand and low clay content likely facilitate spread of underground stems and roots (Herben and Klimešová, 2020; Meller et al., 2022). This increased competitiveness and ability to spread and increased competitiveness could potentially result in rhizomatous species having wider geographic ranges than non-rhizomatous species. On the other hand, investing in belowground organs could also come at a cost of fecundity and sexual reproduction (Eckert, 2002; Vallejo-Marín et al., 2010); while it could help species to persist in the environment, would not necessarily lead to substantial increases in range size.

A second functional group within geophytes are species with underground storage organs (USOs) storing resources such as water and/or non-structural carbohydrates in specialized organs such as tubers (derived from stems or roots), corms (derived from stems), bulbs (derived from leaves), or swollen hypocotyls (derived from the stem region below the first cotyledon but above the radicle or root; Tribble et al., 2021b.) USOs have been suggested to be more common in environments where plant growth is limited by nutrient and moisture availability such as areas with dry and poor soils in the tropics (Jónsdóttir and Watson, 1997; van Groenendael et al., 1997; Prescott et al., 2020). Other examples of environments requiring survival and persistence through drought and cold stress can also be found in temperate environments with marked climatic seasonality and reduced growing seasons, resulting in strategies maximizing photosynthetic and reproductive opportunities (Howard and Cellinese, 2020; Prescott et al., 2020). Seasonal resource storage is a demanding of plant resources, and hence is expected to be more common in environments with strong seasonality but little disturbance, providing sufficient time to build and maintain USOs (Bellingham and Sparrow, 2000).

Only a handful of studies have tested the links between geophytes, seasonality and disturbance in an evolutionary context (e.g., Parsons and Hopper, 2003; Procheş et al., 2006; Cuéllar-Martínez and Sosa, 2016; Sosa and Loera, 2017). One study has found clear evidence for the repeated independent evolution of geophytic growth forms as an adaptation to fire in tree relatives across the savanna biome in Africa (Maurin et al., 2014). However, another study testing for niche shifts in Liliales found no differences in the climatic optima of geophytic versus non-geophytic species (Tribble et al., 2022). Using a phylogenetic comparative approach, Howard et al. (2019) examined whether abiotic climatic stressors, such as temperature and precipitation, were correlated to the evolution of different underground organs, including rhizomes, bulbs, corms, and tubers across all monocots, and found that geophytes tended to occur in environments with stronger temperature seasonality than non-geophytes. They study however did not detect ecological differences among different groups of underground storage organs, potentially due to functionally diverse underground organs coded across tens of families clustering non-homologous structures into single categories (Howard et al., 2019; Howard et al., 2020). Lineage-specific studies have avoided problems with homology as it is easier to classify organs into functionally homologous groups, allowing characterization of climatic drivers related to the evolution of geophytic species in a more realistic way (Evans et al., 2014; Sosa et al., 2016).

Here, we test whether the evolution of underground organs has enabled plants to adapt to specific environments in the large, globally distributed genus *Solanum* L. Specifically, we ask whether geophytic plants occupy similar ecological niches to non-geophytes, and whether species with distinct functional types of underground organs (rhizomatous species and USOs) show significant differences in their environmental niches, following the hypothesis that rhizomatous plants would be expected to show higher diversity in highly productive tropical environments with frequent disturbance. In contrast, species with USO are expected to be more common in environments with prolonged climatic disturbance relating to drought and temperature stresses which force plants to dormancy. In addition, we test whether geophytic organs alter the relationship between geographic range and environmental niche breadth as would be expected if these organs have distinct ecological functions related to dispersal, competition, and vegetative reproduction. Our results demonstrate environmental divergence of the two underground organ types in *Solanum*, with rhizomes and USOs each found to occupy distinct disturbance and temperature regimes. This shows that that geophytic organs that represent different strategies for persistence and competitiveness are successful in different environments. and these differences can be detected at macroecological scale using lineage-specific studies.

## 2 Materials and methods

### 2.1 Study system and underground organ definitions

**FIGURE 1:**
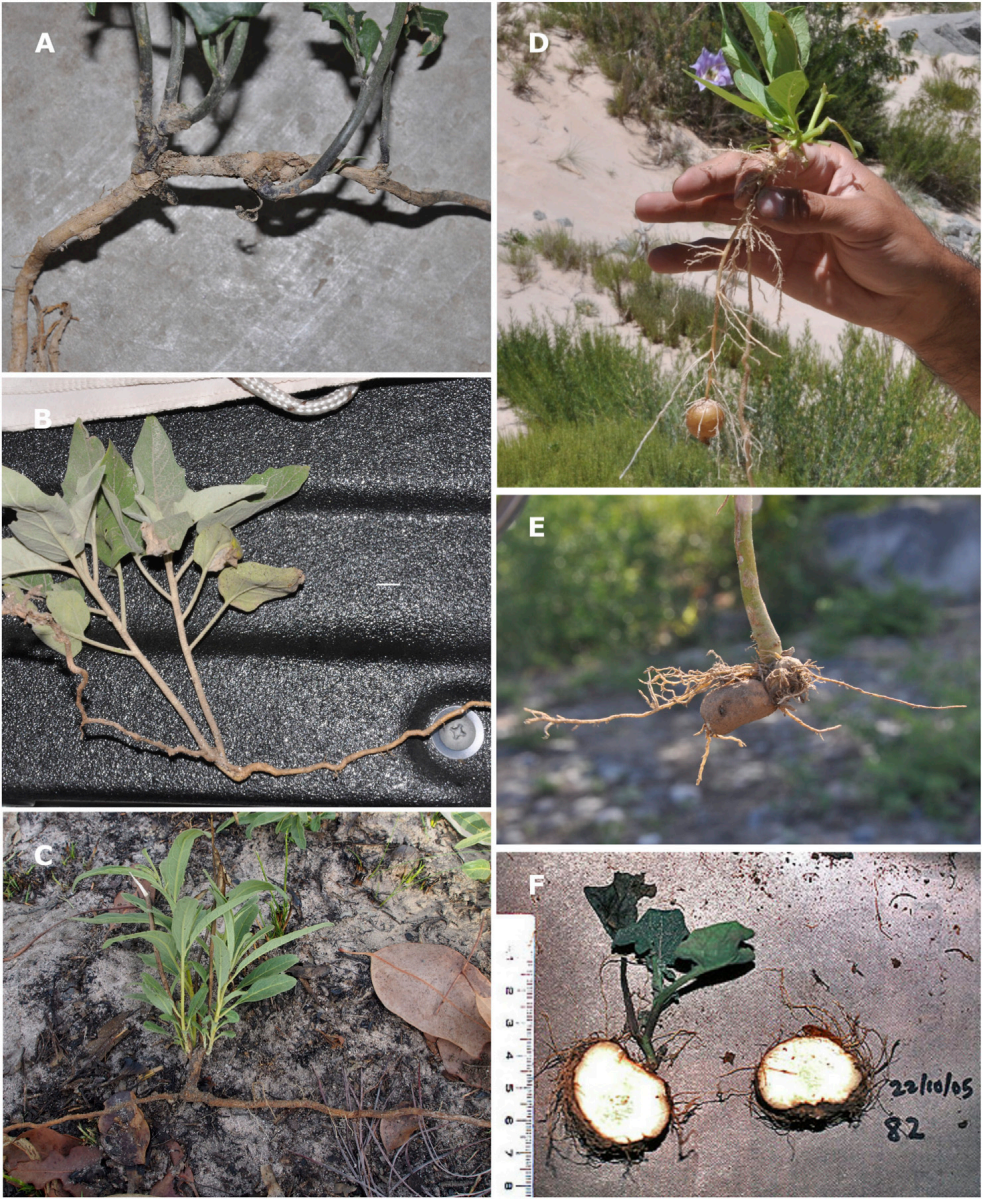
Examples of underground organs observed across different clades of *Solanum.* **(A)** *S. echegarayi* Hieron. (Morelloid clade; photo by S. Knapp); **(B)** *S. reductum* C. V. Morton (Geminata clade; photo by S. Knapp) **(C)** *S. cowiei* Martine (Eastern Hemisphere Spiny clade; photo by C. Martine); **(D)** *S. boliviense* Dunal (Petota clade; photo by S. Knapp); **(E)** *S. hieronymi* Kuntze (Carolinense clade; photo by S. Knapp); **(F)** *S. montanum* L. (Regmandra clade; photo taken at the Lomas de Amancaes, Peru, by S. Knapp).

*Solanum* is an economically important plant genus that includes the cultivated potato (*S. tuberosum* L.). It has served as the model-system for understanding the genetic basis of tuberization in plants, but the genus includes distinct types of underground organs that can be broadly classified into two functional groups: rhizomatous species that focus on lateral spread, and species with USOs focused on starch/water storage. In *Solanum*, USOs are organs that are conspicuously specialized for storing complex carbohydrates and/or water, and include stem tubers in the wild potatoes in *Solanum* section *Petota* (Spooner et al., 2016; Spooner et al., 2019; Figure 1D), root tubers in the Asterophorum (Gouvêa and Stehmann, 2019) and Carolinense clades (Wahlert et al., 2015; Figure 1E), and swollen hypocotyls in the Regmandra clade (Bennett, 2008; Figure 1F). The principal function of these USOs is the storage of different sugars, nutrients and water needed by the plant to undergo dormancy and/or to allow the plant to recover from disturbance.

Rhizomatous species in *Solanum*, in contrast, have belowground stems that enable lateral spread, from which both above ground stems and roots arise (Serebrjakov and Serebrjakova 1965; CLO-PLA database trait definition (Klimešová et al., 2017); Figures 1A, B). Our definition of “rhizomatous” also includes species known as “root sprouters” or “root suckers”; the latter have horizontal roots with adventitious buds from which a whole plant is able to resprout (Miyazaki and Ito, 2004; Pattison et al., 2019), and which are sometimes also referred to as species with “gemmiferous roots” or with “endogenous buds” (Wahlert et al., 2015; Figure 1C). Distinguishing between species that have true rhizomes and those that are root sprouters is particularly difficult from the literature as these structures are generally not clearly or adequately described (plants are sometimes merely referred to as “clonal”), and difficult to observe on herbarium specimens. In both cases, however, these underground horizontal structures in *Solanum* facilitate lateral and clonal dispersal, as well as enabling plants to resprout from disturbance events.

A list of all 1,232 currently accepted species for the genus *Solanum* was obtained from the expertly curated database Solanaceae Source (SolanaceaeSource.org, November 2020). Trait data were derived from species descriptions in taxonomic monographs and from herbarium specimens. All species were categorized as either non-geophytic (931 species, 75% of the total), rhizomatous (180 species, 15% total), or with USOs (121 species, 10% of the total; Supplementary Table S1; Supplementary Datasheet S1). A total of 301 *Solanum* species (24% of the total) were found to be geophytic.

In *Solanum* some species possess both underground storage organs and rhizomatous structures. This includes some species of the Carolinense clade, root sprouters that sometimes also possess root tubers (Wahlert et al., 2015). Nearly all members of the Petota clade have underground rhizomes, which are frequently referred to as “stolons,” a term which in the functional root trait literature usually refers to above-ground lateral stems (Klimešová et al., 2019) but which has been applied to underground rhizomes connecting tubers in the past (Bell and Tomlinson, 1980). Rhizomes in tuberous *Solanum* species show considerable variation in length, varying from moniliform with tubers formed in a string along a short rhizome to species with >1 m long rhizomes with few large tubers at the end (Spooner et al., 2004). Here, we treat all these tuberous *Solanum* species with rhizomes under the category of USOs and consider them distinct from rhizomatous species because the latter do not store large amounts of carbohydrates or water. Some species considered rhizomatous have been shown to have carbohydrate reserves, such as the “tuberized” rhizomes with thickened cortical walls found in *S. elaeagnifolium* and its relatives (Knapp et al., 2017), but they represent a very different system of carbohydrate storage compared to the localized and concentrated starch storage found in USOs such as the stem tubers of potatoes. The categories we use here for USOs and rhizomes may differ from how these terms may be applied in other groups (e.g., Chomicki, 2013) but allow us to explore our questions in *Solanum*.

To account for the phylogenetic relatedness between the study species, we used a recently published species-level phylogeny that included 742 *Solanum* species (60% of those currently accepted; (Gagnon et al., 2022). Cultivated species (*n* = 19) where wild populations are not known or clearly labelled as cultivated were excluded because their distributions reflect anthropogenic commensalism rather than evolutionary patterns related to environment (*S. tuberosum*, *S. lycopersicum, S. melongena, S. muricatum, S. aethiopicum, S. macrocarpon, S. lasiocarpum, S. betaceum, S. sessiliflorum, S. quitoense, S. scabrum, S. aviculare, S. crispum, S. laciniatum, S. laxum, S. pseudocapsicum, S. seaforthianum, S. wendlandii, S. mammosum*). Species used by humans, but which are also known to occur in the wild and where wild populations can clearly be distinguished were included (e.g., *S. torvum*), but we took care to exclude all cultivated records.

### 2.2 Occurrence data

We downloaded taxonomically verified occurrence data for all input species from specimen records in SolanaceaeSource (accessed 21st of April 2021; 115,496 occurrence records) and the Australian Virtual Herbarium (29,305 occurrence points; 5th June 2019, https://doi.org/10.26197/5cf786115b9ef). Occurrence data were cleaned using several steps implemented in R (v. 4.1.0 (R Core Team, 2021), to remove specimens 1) unidentified to species, or that were considered as ornamental or crop species (see section *Phylogeny* below); 2) indicated as cultivated in their label data; 3) lacking latitude/longitude data; 4) with erroneous coordinates (seconds greater than 59 in value); 5) in the sea or large bodies of water; 6) with imprecise coordinates (seconds missing for both latitude and longitude); 7) where coordinates did not match with the country given in label data. In addition, the R package “CoordinateCleaner” (Zizka et al., 2019) was used to remove duplicate specimens, specimens with equal lat/long coordinates, specimens with coordinates corresponding to zero latitude or longitude, as well as specimens that fall within 1 km of a list of c. 10,000 biodiversity institutions. Maps were also generated and visualized to ensure that distributions corresponded to known distributions described in the taxonomic literature, removing aberrant points. This resulted in a dataset with 80,525 occurrence records for 1,169 species (94.8% of all *Solanum* species); 217 out of 224 geophytes were kept in this dataset (98%, see Supplementary Table S1). Average and median number of occurrence records for each geophytic category is summarized in Supplementary Table S1, and 77% of all the retained species in *Solanum* had >5 occurrence records. Spatial filtering was done for all species with >5 records to reduce collection bias and outlier effects in the dataset, where we ensured that there was at least 10 km distance in between occurrence records within the same species.

### 2.3 Environmental data

We selected eight environmental variables with a strong impact on the global distribution of geophytic plants with focus on heat, frost, temperature seasonality, precipitation seasonality, drought, fire, soil sand content, and topography (Prentice et al., 1992). Heat was represented by maximum temperatures of warmest month (bio5), minimum temperatures of coldest month as a proxy for cold stress (bio6), temperature seasonality by temperature annual range (bio7), precipitation seasonality by coefficient of variation (bio15), and drought by the annual Moisture Index (MI; the ratio of annual precipitation to annual potential evapotranspiration). All temperature and precipitation variables were derived from CHELSA climate data at a 30 arcsec spatial resolution (c. 1 km). MI was calculated using the Priestly-Taylor formulation provided by the SPLASH algorithm of (Davis et al., 2017), because it has been shown to perform substantially better in reproducing eddy-flux measurements of actual evapotranspiration under unstressed conditions compared to Penman-Monteith formula (Maes et al., 2018). MI values lower than one indicate there is less precipitation than evapotranspiration, resulting in drought stress for plants thereby providing more information than values solely based on precipitation.

Fire was included as an environmental variable by using the 95th quantile of fire size (q95size), based on a global map calculated by Archibald et al. (2013), at a 0.5° spatial resolution (∼55 km). Fire size is one of the five key characteristics of fire regimes. Inherent challenges of calculating environmental variables related to fires that occurred more than 50 years ago resulted in a large amount of missing data in the q95size layer, so we used custom R scripts to attribute the value of the closest cell if the occurrence point was less than 55 km away from a neighboring cell. Variation in soil density was included by including the proportion of sand particles (>0.05 mm) in the fine earth fraction at a depth of between 0–5 cm from SoilGrids 2.0, at 30 arcsec resolution (Poggio et al., 2021). Soils with higher sand content are hypothesized to allow easier growth and expansion underground for rhizomes (Herben and Klimešová, 2020). Finally, topographic complexity was measured using the vector ruggedness metric (VRM) at 30 arcsec resolution (Amatulli et al., 2018), to test whether USOs and rhizomatous species show differential responses to complex mountainous terrain, where rhizomes would be expected to dominate in flatter terrains with looser soils where underground lateral expansion might be easier. VRM varies from 0 for flat surfaces, to 1 for the most rugged regions (Amatulli et al., 2018).

For the ecological analyses, we only retained occurrence records for which values of all environmental layers were available, resulting in a final dataset with 47,083 records, representing 1,151 species of *Solanum* (Supplementary Table S1). This dataset was used in the PCA, PERMANOVA, Phylogenetic ANOVAs, and for the calculation of niche breadth.

### 2.4 Environmental analyses

#### 2.4.1 PCA with kernel density estimates

We carried out principal component analysis (PCA) using the “factoextra” package using the cleaned occurrence records of *Solanum* (Kassambara and Mundt, 2020). A circle of contribution was used to examine correlation amongst variables, and arrows were colored according to their contributions (in percentage) to all the principal components of the environmental space. All environmental variables had relatively low levels of correlation (0.05–0.70; Supplementary Table S2). We also used the “corrplot” package (Wei and Simko, 2021) to visualize the quality of representation values of all variables for the principal components. Occurrence records of each underground organ category were visualized as kernel density estimates in this PCA space using the methods and scripts as described in (Díaz et al., 2016). Three variables were log-transformed prior to analysis (Supplementary Figure S1).

#### 2.4.2 PERMANOVA

Permutation multivariate analyses of variance (PERMANOVA) were run to test for significant differences in the environmental space between the two geophyte categories and non-geophytes, with a null hypothesis that the centroids and dispersion of the groups were equivalent. We conducted *post hoc* pairwise comparison tests to identify which categories differed significantly from each other. The mean value of each environmental variable was calculated for all 1,151 species, followed by a calculation of a Bray-Curtis distance matrix. We used the function “Adonis2” in the package “vegan” (Oksanen et al., 2022), with 1,000 permutations for the PERMANOVA test. The *R* ^2^ value was used to determine the proportion of the variation that could be explained by the independent variable.

#### 2.4.3 Phylogenetic ANOVA

Phylogenetic ANOVA tests were carried out to identify significant differences between non-geophytes and geophytes along each of the environmental variables. We corrected for non-independence of species using a matrix of expected variance and covariance of residuals based on our phylogenetic tree and the Brownian motion model of evolution, using the function vcv.phylo of the package “ape” (Paradis and Schliep, 2019). Because results from the ancestral state reconstruction analysis suggested a significant clade aggregation of underground organs, we performed the phylogenetic ANOVA by randomizing residuals in a permutation procedure (RRPP) (Adams and Collyer, 2018), using the function lm.rrpp, in the R packages “geomorph” and “RRPP” (Collyer and Adams, 2018; Baken et al., 2021). No difference in the means of environmental variables between groups when accounting for phylogenetic relationships is our null hypothesis. A pairwise comparison test was performed to determine which categories differ significantly from each other. We calculated the mean of each environmental variable for each of the 702 species present in both the environmental dataset and phylogeny (Supplementary Table S1). The test was also carried out on all the eight environmental variables together. We used the same dataset to carry out a PCA and phylogenetic ANOVA test on the first three PCA axes.

### 2.5 Range size and niche breadth

We calculated range sizes using the package “ConR” (Dauby et al., 2017) with alpha hull values (*α* = 2) for spatially non-filtered occurrence data based on Baldaszti (2021). Range sizes could not be calculated for 116 species that had fewer than 2 occurrence records; these were removed from all analyses. Our final dataset for range size and niche breadth included 1,062 species of *Solanum* with 782 non-geophytic and 280 geophytic species (Supplementary Table S1). Niche breadth was calculated using spatially filtered dataset as the sum of the scaled difference of the maximum and minimum value of all eight environmental variables. For species with >10 occurrence records, a subsampling process was introduced to reduce the impact of climatic outliers following Baldaszti (2021).

Prior to analyses, both range size and niche breadth were log-transformed to meet assumptions of normality required for *post hoc* analyses. To determine whether mean range sizes and niche breadth were significantly different among the three groups (e.g., non-geophytes, species with USOs, and rhizomatous species), we carried out a non-parametric Kruskal–Wallis test using the package “rstatix” (Kassambara, 2021). In addition, *post hoc* pairwise comparisons between the three groups were carried out to determine which pairs were significantly different from one another, using the Dunn test with Bonferroni adjustment.

We examined the correlation between climatic niche breadth (within the individual climatic variables and overall) and range size using a linear model with log-transformed values for both range size and niche breadth. The “smart” package (Warton et al., 2012) was used to determine whether the relationship between range size and niche breadth across the three growth form groups (USOs, rhizomes and non-geophytes) were similar. Statistical tests from the “Standardized Major Axis Tests and Routines” (SMATR) allowed us to determine whether there were any significant differences in the slope, as well as shifts in elevation or along the *x*-axis in the linear models calculated for each growth form separately. We also used robust T and adjusted *p*-values for multiple comparison tests. We calculated 95% confidence intervals for each slope by carrying out 1,000 bootstrap replicates using a custom R script.

### 2.6 Evolutionary analysis

Ancestral trait reconstruction analysis was run using stochastic character mapping (SIMMAP) to examine the evolutionary lability of the underground organs across the phylogeny of *Solanum* using the “phytools” package (Revell, 2012). We treated underground organ trait data as unordered and equally weighted for all three categories. A likelihood ratio test with the Akaike Information Criterion was used to identify the best fitting transition rate model: equal transition rate among characters states (ER), all rates different model (ARD), and symmetrical rate model (SYM). Based on the likelihood ratio test and the AICc criterion, we found the ARD model had the best fit. A total of 200 simulations were run to obtain a posterior probability distribution of ancestral states across the phylogeny with 702 tips kept for which environmental data were available.

## 3 Results

### 3.1 Environmental niche of geophytes

#### 3.1.1 Geographical and ecological patterns of geophyte diversity

**FIGURE 2:**
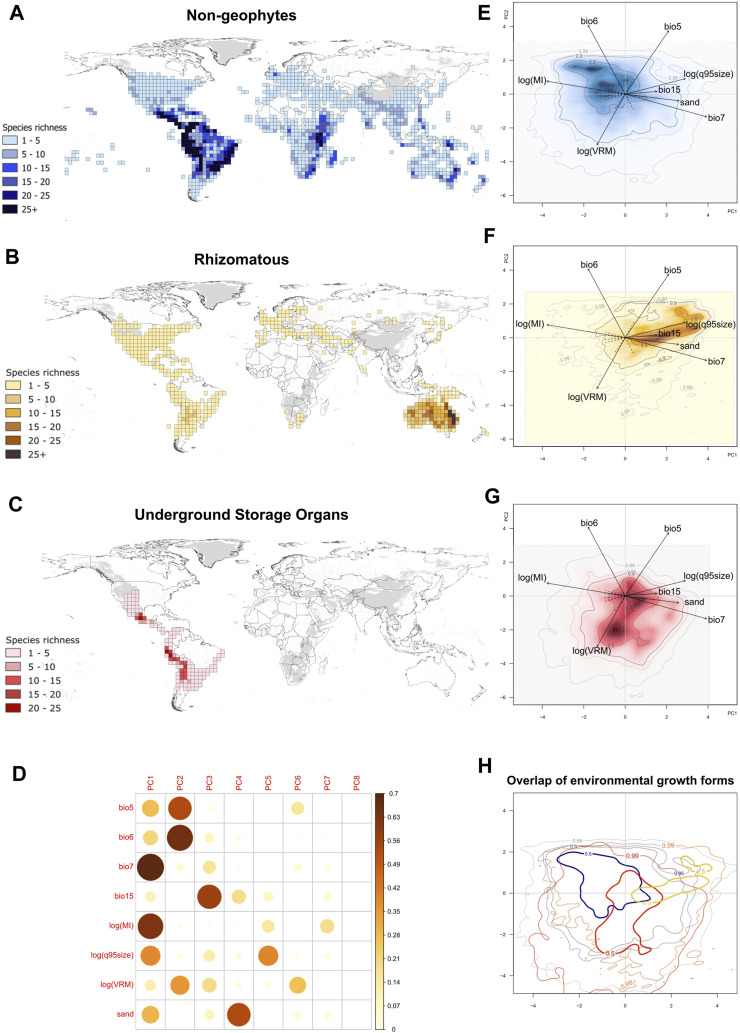
Distribution of geophytic and non-geophytic *Solanum* species in geographical and ecological space. **(A)** Map of species richness of non-geophytic species; **(B)** Map of species richness of rhizomatous species; **(C)** Map of species richness of species with USOs; **(D)** correlation matrix of cos^2^ variables to each environmental axis; **(E)** PCA kernel density estimate of non-geophytic species; **(F)** PCA kernel density estimate of rhizomatous species; **(G)** PCA kernel density of species with USOs; **(H)** overlap of the kernel density estimates from all three growth forms in PCA space, with the 50th quantile line illustrated in bold. Species richness maps were generated using the occurrence record dataset used to generate the PCA kernel densities (47,083 occurrence records). Kernel density estimates have color gradients highlighting the regions of highest and lowest growth form occurrence probability. In addition, contour lines correspond to the 0.5, 0.95, and 0.99 quantiles of the respective probability distribution were added. Definition of environmental variables: bio5, maximum temperature of the warmest month; bio6, minimum temperature of the coldest month; bio7, temperature seasonality; bio15, precipitation seasonality; log(MI), log of moisture index, representing drought; log(q95size), log of 95th quantile for fire size; log(VRM), log of vector ruggedness metric, for topographic heterogeneity; sand, proportion of sand particles (>0.05 mm) in the fine earth fraction at a depth of between 0–5 cm.

Mapping the species richness of geophytes in *Solanum* reveals distinct geographic distribution patterns between geophytes and non-geophytes as well as between two geophytic groups (Figures 2A–C). Rhizomatous species display an amphitropical distribution with greatest diversity in Australia (Figure 2B). In contrast, species with USOs are restricted to South and Central America, with species richness concentrated in high-elevation habitats (Figure 2C). Differences between the geophytes are also clearly visible in environmental space, where the first three axes of the PCA explained 70% of the variation in the data (Figure 2; Supplementary Figure S2). The first PC axis (33.9%) was most strongly correlated with seasonality of temperature (bio7), as well as drought (log(MI)) and sand, and the second PC axis (20.3%) was most strongly correlated with maximum temperature (bio5), minimum temperature (bio6), and topographic heterogeneity (log(VRM); Figure 2D). The third axis, which explained 15.8% of the variation of the data, was strongly correlated with precipitation seasonality (bio15) and fire size (log(q95size); Figure 2D; Supplementary Figure S2).

We observed differences in the occupancy of the 50% kernel density estimate of non-geophytic, rhizomatous, and USO species in ecological space, with non-geophytic growth forms occurring predominantly in environments with less precipitation and temperature seasonality, as well as less drought and with higher minimum temperatures (Figures 2E–H). In contrast, the 50% kernel density estimate of rhizomatous species was in environments with higher values of temperature and precipitation seasonality (bio7 and bio15), low values of MI (more drought) and high values of absolute maximum temperature (bio5) (Figures 2E–H). Rhizomatous species also occurred in environments with larger fire sizes and in soils with higher sand content (Figures 2E–H). The 50% kernel density estimate of USO species was in environments that have lower absolute minimum temperatures (bio6) (Figures 2E–H).

#### 3.1.2 PERMANOVA

The PERMANOVA analysis on all three groups led to a rejection of the null hypothesis (*R* ^2^: 0.11, Table 1), as did the three *post hoc* pairwise comparisons (Table 1), indicating significant differences in the environmental space between the two geophyte categories and non-geophytes, where the centroids and dispersion of the groups were not equivalent. The differences were most significant between rhizomatous and USO species, as evidenced by the highest *R* ^2^ value (*R* ^2^ 0.28, Table 1).

**TABLE 1:** Results from the PERMANOVA tests, the SMATR tests in slope, elevation and shift, and results from Kruskal–Wallis tests comparing range size and niche breadth across the three different growth forms. *p*-values below 0.05 are highlighted in bold; *p*-values equal or below 0.01 are accompanied by an asterisk.

PERMANOVA comparison											
Comparison	*p*-value		*R* ^2^								
All growth forms	0.001*		0.19								
NG vs. RH	0.001*		0.16								
NG vs. USO	0.001*		0.07								
RH vs. USO	0.001*		0.28								

SMATR tests											
						Pairwise comparisons					
						NG vs. RH		NG vs. USO		RH vs. USO	
Niche breadth ∼ log (range sizes)	*p*-value		Test Stat			*p*-value	Test Stat	*p*-value	Test Stat	*p*-value	Test Stat
SMATR—slope	0.002*		11.65			0.002*	11.23	0.97	0.09	0.06	5.42
SMATR—elevation	<0.001*		61.19			<0.001*	52.57	0.18	3.37	<0.001*	50.08
SMATR—shift	0.66		0.83								

Kruskal–Wallis tests											
						Pairwise comparisons: Dunn test					
						NG vs. RH		NG vs. USO		RH vs. USO	
	*p*-value	Test stat			Eta^2^	P.adj-value	Test stat	P.adj-value	Test stat	P.adj -value	Test stat
Range size	0.003*	11.4			0.009	0.007*	3.05	1	−0.965	**0.011**	−2.91
Log Niche breadth	0.11	4.34			0.002						

NG, non-geophytic species; RH, rhizomatous species; USO, species with underground storage organs.

#### 3.1.3 Phylogenetic ANOVA

Phylogenetic ANOVAS revealed significant differences in mean averages between the groups when testing on the first three PCA axes, with rhizomes being significantly different from non-geophytic and USO species (Table 2). In addition, phylogenetic ANOVAS conducted along each of the eight environmental variables were significant (Table 2). Subsequent pairwise comparison tests showed significant differences between all three groups for bio5 and bio7, related to maximum annual temperature and temperature seasonality, whereas geophytes where significantly different from non-geophytes for variables related to drought (log(MI)) and minimum temperature (bio6; Table 2). Rhizomatous species differed significantly from USO and non-geophytic species for the variables related to fire (log(q95size)), soil (sand) and topographic heterogeneity (log(VRM); Table 2). Finally, USOs differed significantly from rhizomatous and non-geophytic species for precipitation seasonality (bio15) and topographic heterogeneity (log(VRM); Table 2).

**TABLE 2:** Results from the phylogenetic ANOVA tests conducted with LM-RRPP, with subsequent pairwise comparisons. *p*-values below 0.05 are indicated in bold; *p*-values equal or below 0.01 are indicated with asterisks.

Environmental variable	LM-RRPP			Pairwise comparisons									
				NG-RH			NG-USO			RH-USO			
	*p*-value	F	*R* ^2^	*p*-value	Z	d	*p*-value	Z	d	*p*-value	Z	d	Conclusion
All 8 variables	0.001*	78.80	0.18	0.001*	5.95	211.00	0.509	0.01	13.02	0.001*	5.34	224.02	RH different
bio5	0.001*	49.01	0.12	0.001*	4.93	40.41	0.001*	3.83	30.98	0.001*	5.81	71.39	All three different
bio6	0.001*	44.52	0.11	0.001*	3.90	33.26	0.001*	4.79	53.50	0.02	1.93	20.23	RH + USO are different from NG
bio7	0.001*	83.64	0.19	0.001*	6.13	73.65	0.002*	2.61	22.51	0.001*	3.97	51.14	All three different
bio15	0.001*	20.03	0.05	0.397	0.35	2.53	0.001*	4.03	18.26	0.001*	3.61	20.79	USO different
log(MI)	0.001*	24.55	0.07	0.001*	4.16	0.12	0.001*	3.04	0.09	0.297	0.62	0.03	RH + USO are different from NG
log(q95size)	0.001*	56.94	0.14	0.001*	5.59	1.26	0.618	−0.29	0.07	0.001*	4.39	1.19	RH different
log(VRM)	0.001*	21.32	0.06	0.001*	4.28	0.002	0.20	0.90	0.0005	0.001*	3.78	0.003	RH different
sand	0.001*	75.88	0.18	0.001*	5.83	131.58	0.018	1.95	30.70	0.001*	4.39	100.88	RH different
PC1+PC2+PC3	0.002*	7.86	0.02	0.001*	2.91	1.13	0.892	−1.29	0.14	0.342	0.43	0.99	RH different from NG
(70.0%)													
PC1 (33.9%)	0.001*	9.76	0.03	0.001*	3124	0.81	0.735	−0.66	0.22	0.40	0.38	0.59	RH different from NG
PC2 (20.3%)	0.844	0.17	0.0005										No differences
PC3 (15.8%)	0.174	1.67	0.005										No differences

NG, non-geophytic species; RH, rhizomatous species; USO, species with underground storage organs. Definition of environmental variables: bio5, maximum temperature of the warmest month; bio6, minimum temperature of the coldest month; bio7, temperature seasonality; bio15, precipitation seasonality; log(MI), log of moisture index, representing drought; log(q95size), log of 95th quantile for fire size; log(VRM), log of vector ruggedness metric, for topographic heterogeneity; sand, proportion of sand particles (>0.05 mm) in the fine earth fraction at a depth of between 0–5 cm.

### 3.2 Range size and niche breadth

Significant differences between the means of range sizes were detected with the Kruskal–Wallis test, with rhizomatous species having larger range sizes than all other species (Table 1; Supplementary Figure S3). While the SMATr analysis was not significant for the shift test, it did identify significant differences in the slope and in the elevation of the linear models of the three groups, with rhizomatous species having a slightly stronger correlation between range size and niche breadth than non-geophytic and USO species (Figure 4; Table 1). Finally, no significant differences in niche breadth were found between the three groups (Table 1; Supplementary Figure S3).

### 3.3 Evolutionary lability of growth forms

Ancestral trait reconstruction analyses indicated distinct patterns of underground organ evolution across *Solanum,* with frequent origins of rhizomatous species and only occasional origins of USOs (Figure 5). Rhizomatous species evolved on average 28 times, and reversals back to non-geophytic growth forms were observed on average 26 times (Figure 5). In contrast, USOs evolved on average 5 times across the entire phylogeny of *Solanum,* with no reversals back to a non-geophytic growth form (Figure 5). No transitions between rhizomatous and USO species were observed (Figure 5).

## 4 Discussion

**FIGURE 3:**
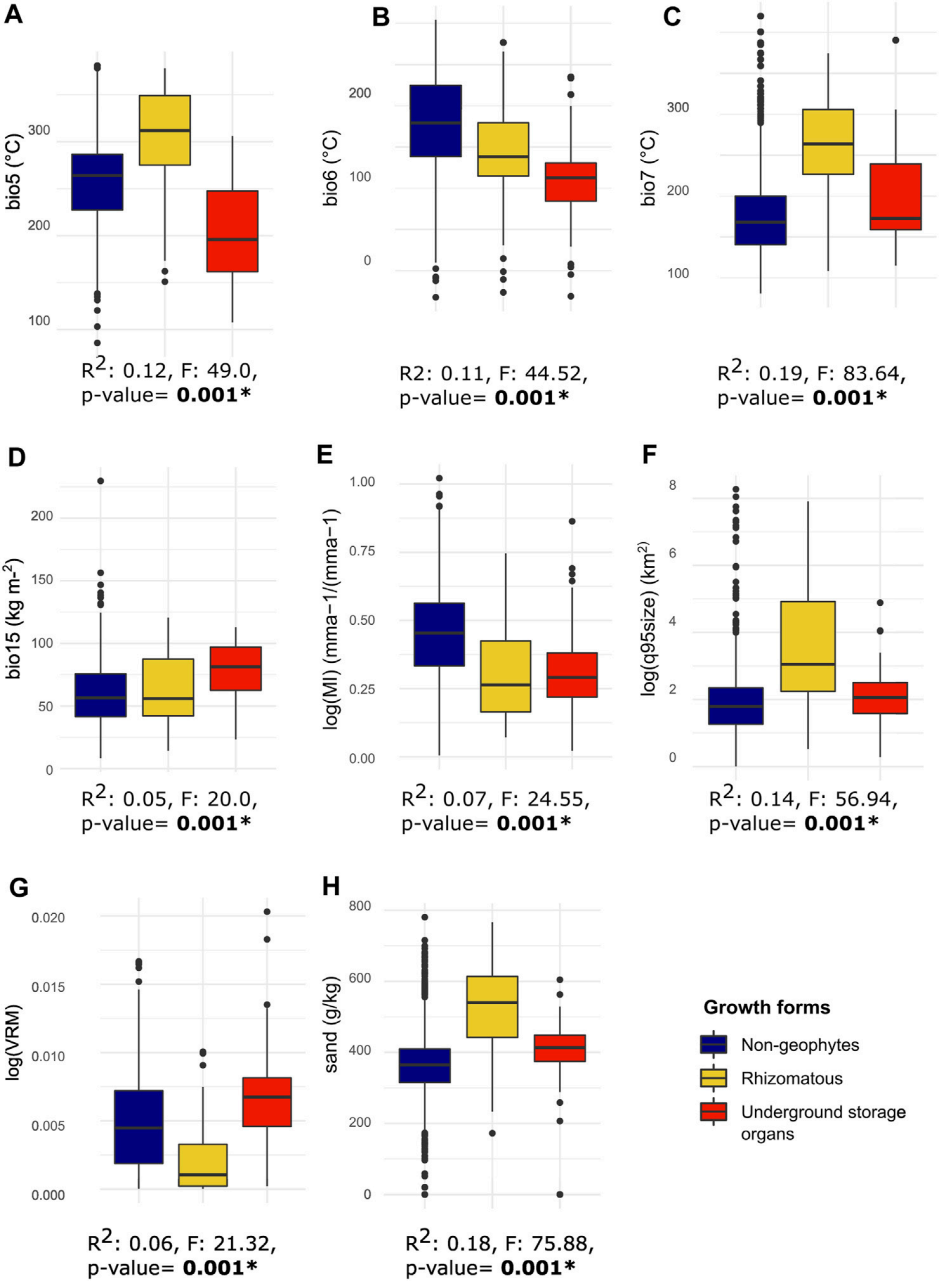
Boxplots and LM-RRPP results for the eight environmental variables, grouped according to our three growth forms. Below each boxplot, results from the LM-RRPP phylogenetic ANOVA tests are reported, with the R2 values, F values and resulting *p*-value. **(A)** bio5, maximum temperature of the warmest month; **(B)** bio6, minimum temperature of the coldest month; **(C)** bio 7, temperature seasonality; **(D)** bio15, precipitation seasonality; **(E)** log(MI), drought, or log of moisture index; **(F)** log(q95size), log of 95th quantile of fire size; **(G)** log(VRM), topographic heterogeneity or log of vector ruggedness metric; **(H)** sand, proportion of sand particles (>0.05 mm) in the fine earth fraction at a depth of between 0–5 cm. Chelsa climate variables are scaled by 0.1.

**FIGURE 4:**
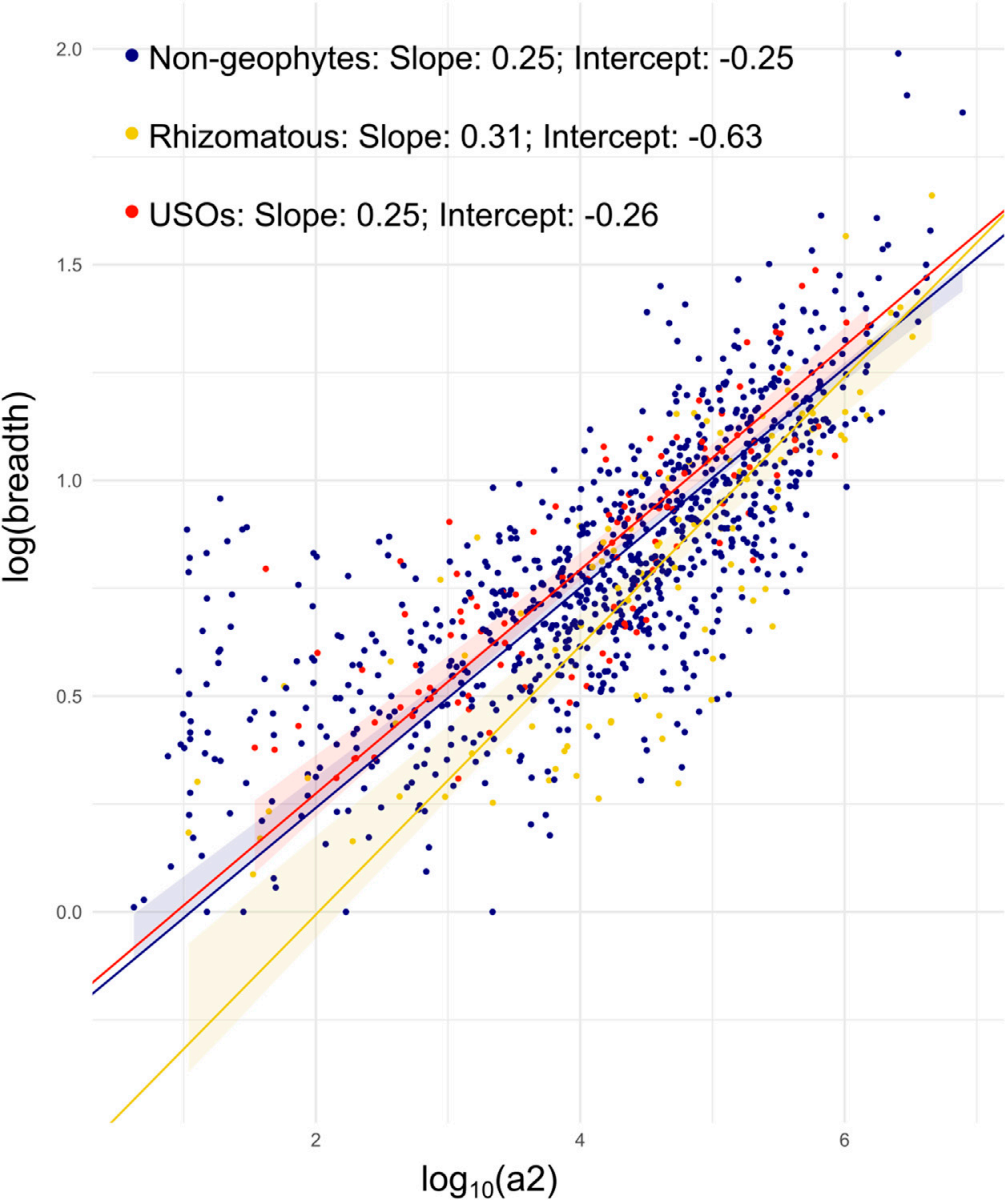
Results from SMATr analysis. Scatterplot of range size against niche breadth, with different labels for growth forms, and SMAs included; 95% confidence intervals for each slope by carrying out 1,000 bootstrap replicates; color legend for lines is as follows: blue, non-geophytes; yellow, rhizomatous species; red, species with USOs.

**FIGURE 5:**
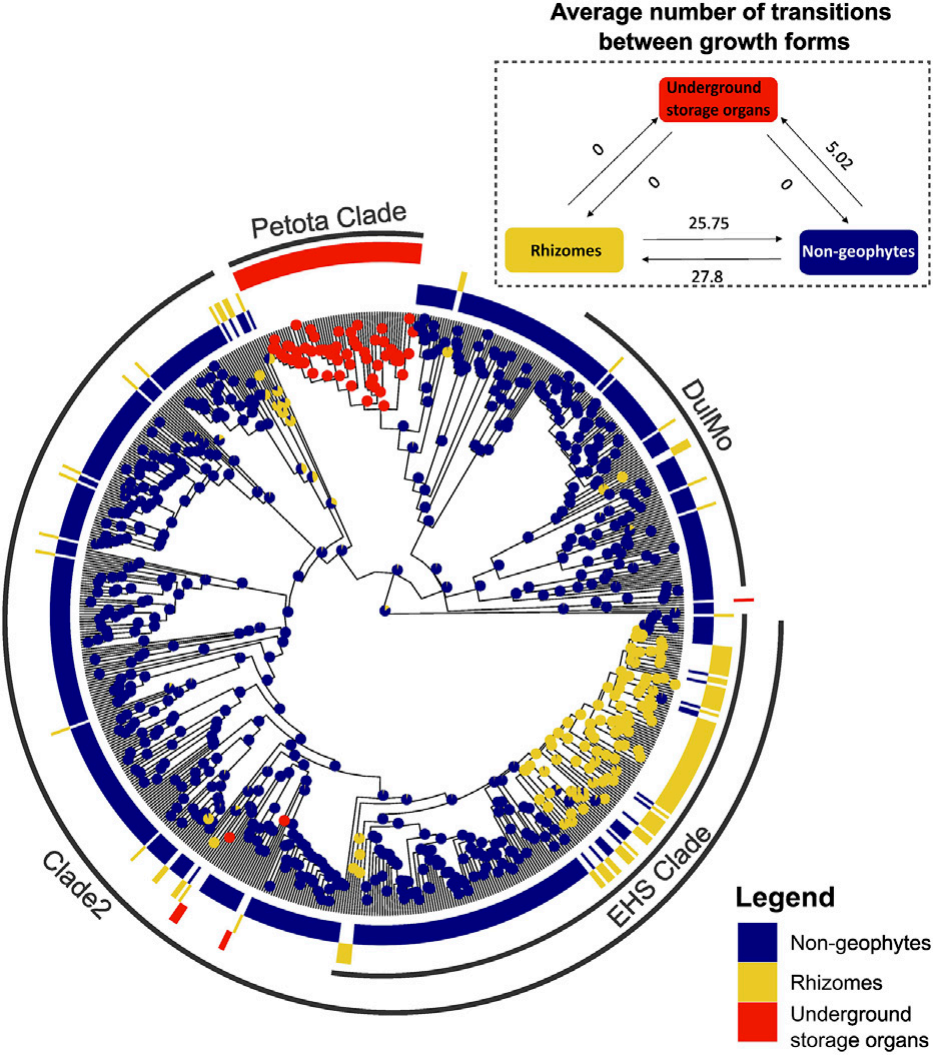
Ancestral trait reconstruction of geophytic and non-geophytic growth forms, using SIMMAP.

Our analysis of geophyte evolution within a single, morphologically diverse, species-rich, and globally distributed genus found different patterns of species richness for non-geophytes and geophytic species (Figure 2). It also showed a strong correlation of different underground organs with climatic, edaphic, and topographic factors in *Solanum,* when accounting for the phylogenetic relationships between species (Figures 2, 3; Table 2). While there are differences in the range size of species with different growth forms, there are not differences in niche breadth (Figure 4; Table 1; Supplementary Figure S3). Finally, we show differences in the evolutionary lability of these traits (Figure 5), which has implications about how easy it is to evolve and adapt to habitats characterized by different types of environmental disturbances.

### 4.1 Divergence of geophytes and non-geophytes

Our results confirm previous findings (Howard et al., 2019) that the evolution of geophytic growth forms is strongly associated with temperature seasonality, but we also find association with drought (Figure 2; Supplementary Figure S2) with significant differences seen between geophytic and non-geophytic species (Tables 1, 2; Figure 2). Geophytic *Solanum* species generally occur in environments that are more prone to drought and seasonal disturbance, as well as with more extreme minimum and maximum annual temperature (Figure 2), in comparison with their non-geophytic congeners (Figures 2E–H). This indicates that geophytic growth forms may act as potential eco-evolutionary enablers for lineages to enter environments dominated by harsh conditions, such as drought and temperature seasonality.

One of the most novel and interesting results in our study is the apparently clear environmental differences between the two distinct types of geophytic organs in *Solanum*. Rhizomatous species are strongly associated with environments that experience severe but less seasonal drought and larger fires. Fires are hugely influential in shaping ecosystem assembly and functional diversity (Pausas et al., 2018), with plants adopting various strategies to overcome, survive and persist different fire regimes, including the ability to resprout from underground bud banks (Pausas et al., 2016). The interplay between drought and fire strongly influences the frequency of resprouting species in the Cape Floristic region, where prolonged drought periods combined with fire favor resprouters (Ojeda, 1998). The presence of both drought and fire may also explain the number of rhizomatous *Solanum* species in Australia (Figure 2). In addition to fire, sandy soils and low topographic complexity are associated with rhizomatous species in *Solanum* (Table 2; Figure 4), likely due to poorly consolidated soils aiding lateral expansion.

In contrast, our results indicate that the evolution of USOs is associated with environments with less severe but more prolonged drought and cold stress (but not frost) and in which fire are not as large or severe. USOs in *Solanum* are mostly found in the large Petota clade and the environmental signal of USOs is hence driven mostly by the strong phylogenetic aggregation in our data. This can be seen in the distribution of species with USOs in American montane regions (Table 2; Figures 2, 4) reflecting species diversity patterns of the Petota clade whose species are concentrated in South America in regions like the Andes, or the Sierra Madre Mountain range in Mexico (Spooner et al., 2004; Spooner et al., 2016; Spooner et al., 2019). The link between USOs and tropical montane regions follows our expectations that USO species have evolved to persist in environments with intermediate levels of disturbance and/or limitations in growth and biomass production. Plant growth in tropical montane regions is not limited by strong seasonal changes in light availability and temperature as it is in high latitude temperate areas, but rather by water availability (including precipitation seasonality, bio15 in Table 2), that can occur due to shifts in elevation or strong rain-shadow effects. Further exploration of functional and morphological diversity of USOs in *Solanum*, especially in smaller clades presenting root tubers and swollen caudices may reveal additional patterns. The swollen caudices found in the Regmandra clade (here defined as USOs) occur in species occurring in dry seasonal environments in the coastal fog-deserts of western coasts of South America (Bennett, 2008), and root tubers are found in members of the Carolinense clade from the Chaco dry woodlands that experience frost (Wahlert et al., 2015). Root tubers in the Asterophorum clade, however, are found in species occurring in damper soil conditions in the Brazilian Atlantic forests (Gouvêa and Stehmann, 2019) in forest edges prone to disturbance.

Another seldom-explored aspect that might influence the distribution of geophytes is the pressure of herbivory (but see Meller et al., 2022). Placements of buds belowground protects meristems and allows plants to resprout after a herbivory event (Ott et al., 2019) involving, for example, mammalian grazing or insect attack on new shoots. In *Solanum*, defences against herbivory can be both mechanical (prickles and stellate hairs) and chemical (alkaloids and terpenoids). These may prove deterrents, but mammals are known to graze on prickly solanums (e.g., Pringle et al., 2014) and specialist herbivores are known to overcome chemical defences (e.g., Brown, 1987). Rhizomatous species of *Solanum* are especially common in the Eastern Hemisphere Spiny (EHS) clade, a group with abundant prickles on leaves and stems (Symon, 1981; Bean, 2004), and studies with the rhizomatous *S. carolinense* of the Carolinense clade (Nihranz et al., 2019) have demonstrated the importance of belowground organs in storing information about environmental stressors such as herbivory. Induced defenses transmitted through rhizomes affect offshoot growth and herbivore resistance, but are compromised by inbreeding (Nihranz et al., 2019), suggesting complex inter-seasonal patterns of plant-animal interaction in at least this rhizomatous *Solanum* species. Leaves in many members of the Petota clade that all have USOs contain high levels of steroidal alkaloids that are likely to play a role in plant defence (Spooner et al., 2004; Spooner et al., 2016; Spooner et al., 2019), perhaps through activation of chemical cascades as has been shown in *Nicotiana* (e.g., Kessler et al., 2010).

### 4.2 Relationship between range sizes, niche shift and niche expansion

The question remains whether underground organs enable niche shifts or whether they expand niches by enabling species to persist and reproduce across a wider range of environmental conditions. Our results show that rhizomatous species have significantly larger mean range sizes (Table 1) than do either non-geophytes or species possessing USOs as defined here, a pattern similar to monocots (Howard et al., 2019). There is also a stronger relationship between range size and niche breadth in rhizomatous species (Figure 4). The increase in range size is not, however, related to increase in overall niche breadth when compared to species with USOs and non-geophytes (Table 1). This pattern could be explained by the fact that rhizomatous species are better able to disperse laterally by vegetative means leading to larger range sizes, but their ecological niche breath is no wider than that of non-rhizomatous species, suggesting that range size is strongly constrained by ecological niche but less so by dispersal.

Niche shifts in geophytes could be facilitated by expansion of niche breadth through exaptation (Gould and Vrba, 1982; Donoghue, 1989). Escape from recurrent episodes of aboveground disturbances such as fire and herbivory through development of underground buds could allow such species to expand into a different set of environments, with different stressors. Fire resistance is thought to have led to the evolution of geoxyle trees in African savannas and thus allowed them to later persist in and invade more frost-prone environments (Davies et al., 2017; Lamont et al., 2017).

### 4.3 Does trait lability reflect differences in evolvability?

We show striking differences in evolutionary lability of underground growth forms in *Solanum*. For example, the rhizomatous habit evolved independently more than 27 times across *Solanum* with the 25 of these occurring in the EHS clade where plants are predominantly root-sprouters. The lability is most apparent when seen in contrast to the conserved nature of USOs, which have evolved in only five independent lineages, with no reversals to a non-geophytic growth form (Figure 5). The lability in the rhizomatous habit has been observed before in other studies across monocots (Howard et al., 2019) and in temperate herbs in eastern Europe (Herben and Klimešová, 2020), whereas USOs are often confined to particular lineages (Howard et al., 2019).

The conserved nature of species with USOs suggests that these organs are difficult to evolve, possibly due to complex set of genomic and molecular processes required for the development of storage tissues in underground environments. Anatomical and molecular mechanisms regulating storage root formation in plants have been particularly well-studied in crop species, including in potatoes [reviewed by Chen and Tang (2017)], and have shown that tuber formation is complex. In potatoes, the key genes involved in tuber initiation have been shown to have evolved as a result of expansion and neo-functionalisation of FLOWERING LOCUS T (FT) proteins, also involved in the initiation of flowering (Abelenda et al., 2011; Navarro et al., 2015). The involvement of FT genes in initiation and formation of USOs have also been found in other distantly related geophytes, including in the Nymphaeales, monocot, and eudicot lineages (reviewed by Tribble et al., 2021a). Comparative transcriptomic studies have also identified other genes that enable USO formation, by allowing the plant to increase storage of water and carbohydrates (e.g., starch biosynthesis, lignin biosynthesis, cell wall modifications; Hearn et al., 2018; Tribble et al., 2021a). In comparison, much less is known about the genetic control of underground rhizomes or the development of adventitious buds on rhizomes. QTL studies in *Sorghum* have shown that rhizomatous phenotypes seem to be controlled by a few genes (Paterson et al., 1995; Yim and Bayer, 1997; Washburn et al., 2013; Hu et al., 2011). There is an increasing interest in the genetic control of rhizomes due to their role in the establishment of perenniality and contributions to weediness in crops (Hu et al., 2011). These factors are also of considerable interest in development of perennial crops from annual progenitors (Kantar et al., 2016; Ciotir et al., 2019; Kong et al., 2022).

Underground structures in *Solanum* are still poorly described relative to aboveground morphology. Plant collectors tend to focus on parts pressable on herbarium sheets, and excavation of tubers or rhizomes is often time-consuming [for an example, see discussion of *S. echegarayi* in Knapp et al. (2023)]. Documentation of underground structures in *Solanum* is expanding with increased field collection and the collaboration between ecologists and botanists in examining plant organs and their functions in the environment. Root tubers in the Asterophorum clade, for example, were only described less than 5 years ago (Gouvêa and Stehmann, 2019), and occur in species that are neither rare nor uncommon. We expect that knowledge of underground structures will evolve as botanists and ecologists better document their occurrence. Our dataset used here is likely to improve with time and collaborative effort.

### 4.4 Conclusion

We demonstrate that in *Solanum,* geophytes not only occupy environments with strong temperature seasonality and drought but show clear functional and ecological diversification between USO and rhizomatous growth forms. Such studies outside monocots are still surprisingly rare and our work provides a unique insight into how underground growth forms can evolve within a globally distributed clade that inhabits a wide range of habitat types.

More in-depth approaches for characterizing the functional ecology of underground organs in *Solanum* is a priority, but current available databases on functional root traits contain little data for this genus, despite its global distribution and agronomic importance. For example, only 12 of 1,232 species of *Solanum* (about 1%) have data available in the Global Root Trait database (GRootT), which seeks to build a large-scale, standardized and curated database of key root traits for plants (Guerrero-Ramírez et al., 2021). The available data is principally limited to traits related to resource acquisition, longevity, and root depth, and provide limited information on how these underground organs allow species to spread, compete and persist in the environment. Ultimately, developing a dataset of underground traits combined with aboveground traits will allow integration of datasets across different plant groups (Howard et al., 2019), and achieve better evolutionary explanatory power. Our dataset for *Solanum*, spanning a wide variety of habitats worldwide, is a significant first step in enabling further phylogenetically relevant macro-ecological and evolutionary studies.

## Data Availability

The original contributions presented in the study are included in the article/Supplementary Material, further inquiries can be directed to the corresponding author.
