# Supplementary material for: Functional and ecological diversification of underground organs in Solanum
Source: Front Genet. 2023 Oct 10;14:1231413. doi: 10.3389/fgene.2023.1231413 (PMC10597785; doi:10.3389/fgene.2023.1231413)

Supplementary Material

# Supplementary Data

**Supplementary Material 1.** Supplementary Figures 1 to 4, and Supplementary Tables 1 and 2.

**Supplementary Material 2*.*** List of 1232 accepted species of *Solanum,* scored according to growth form in our study (non-geophyte, rhizomatous, USOs). References for scoring also provided in separate tab.

***Supplementary Material 3.** Occurrence dataset. List of occurrence records retained for the analysis.

***Supplementary Material 4.** Moisture Index Map, calculated according to the Splash algorithm, at a 30 arcsec spatial resolution (c. 1 km).

File is 961,853 Ko in size, and available at the following link:

https://1drv.ms/i/s!AnL3loahWdw0kJEWnSXKKgTfwM3NZg?e=ik3aOx

*Supplementary Material 3 and 4 are likely to be deposited in an online repository.

# Supplementary Figures and Tables

## Supplementary Tables

## Supplementary Table 1. Characteristics of the occurrence datasets before and after spatial filtering.

| **Category** | **Nb. Species** | **Occurrence points** | **Mean number of occurrence points/species** | **Median number of occurrence points/species** | **Species with <=5 occurrence points** | | **6-30 occurrence points** | **>30 occurrence points** |
| --- | --- | --- | --- | --- | --- | --- | --- | --- |
| **Cleaned occurrence dataset before spatial filtering** | | | | | | | | |
| Non-geophytes | 873 | 49058 | 56.2 | 19 | 177 | | 356 | 340 |
| Rhizomatous | 179 | 22129 | 123.6 | 54 | 21 | | 46 | 112 |
| USOs | 117 | 9338 | 79.8 | 28 | 22 | | 40 | 55 |
| **Total** | 1,169  (94.8%) | 80,525 | 68.9 | 23 | 220 | | 442 | 507 |
| **Cleaned occurrence dataset with spatial filtering, and occurrence records with missing environmental variables removed** | | | | | | | | |
| Non-geophytes | 857 | 30,691 | 35.8 | 14 | 209 | | 410 | 238 |
| Rhizomatous | 177 | 12,243 | 69.2 | 27 | 24 | | 74 | 79 |
| USOs | 117 | 4,149 | 35.5 | 14 | 28 | | 56 | 33 |
| **Total** | 1,151 (93.4%) | 47,083 | 40.9 | 15 | 261 | | 540 | 350 |
| **1232 species of *Solanum*** | | | **702 species from the phylogeny** | | | **1062 species from the niche breadth-range size hypothesis** | | |
| Non-geophytes | 931 |  | Non-geophytes | 527 |  | | Non-geophytes | 782 |
| Rhizomatous | 180 |  | Rhizomatous | 111 |  | | Rhizomatous | 170 |
| USOs | 121 |  | USOs | 64 |  | | USOs | 110 |
| **Total** | 1232 |  | **Total** | 702  (57%) |  | | **Total** | 1062  (86%) |

## Supplementary Table 2. Correlation amongst eight environmental variables used in the PCA analysis.

|  | **Bio5** | **Bio6** | **Bio7** | **Bio15** | **logMI** | **logq95size** | **logVRM** | **sand** |
| --- | --- | --- | --- | --- | --- | --- | --- | --- |
| Bio5 | 1 | 0.338505 | 0.43387 | 0.059779 | -0.28356 | 0.306309 | -0.46297 | 0.176427 |
| Bio6 | 0.338505 | 1 | -0.70091 | 0.014977 | 0.355042 | -0.10856 | -0.10838 | -0.12749 |
| Bio7 | 0.43387 | -0.70091 | 1 | 0.030985 | -0.55489 | 0.336138 | -0.24714 | 0.255824 |
| Bio15 | 0.059779 | 0.014977 | 0.030985 | 1 | -0.30468 | 0.329468 | 0.060077 | 0.134886 |
| logMI | -0.28356 | 0.355042 | -0.55489 | -0.30468 | 1 | -0.31718 | 0.164824 | -0.42709 |
| logq95size | 0.306309 | -0.10856 | 0.336138 | 0.329468 | -0.31718 | 1 | -0.13912 | 0.289677 |
| logVRM | -0.46297 | -0.10838 | -0.24714 | 0.060077 | 0.164824 | -0.13912 | 1 | 0.004255 |
| Sand | 0.176427 | -0.12749 | 0.255824 | 0.134886 | -0.42709 | 0.289677 | 0.004255 | 1 |

## Supplementary Figures

## Supplementary Figure 1. Histograms of variables. a) Before log transformation. b) After log transformation of MI, q95size and VRM.


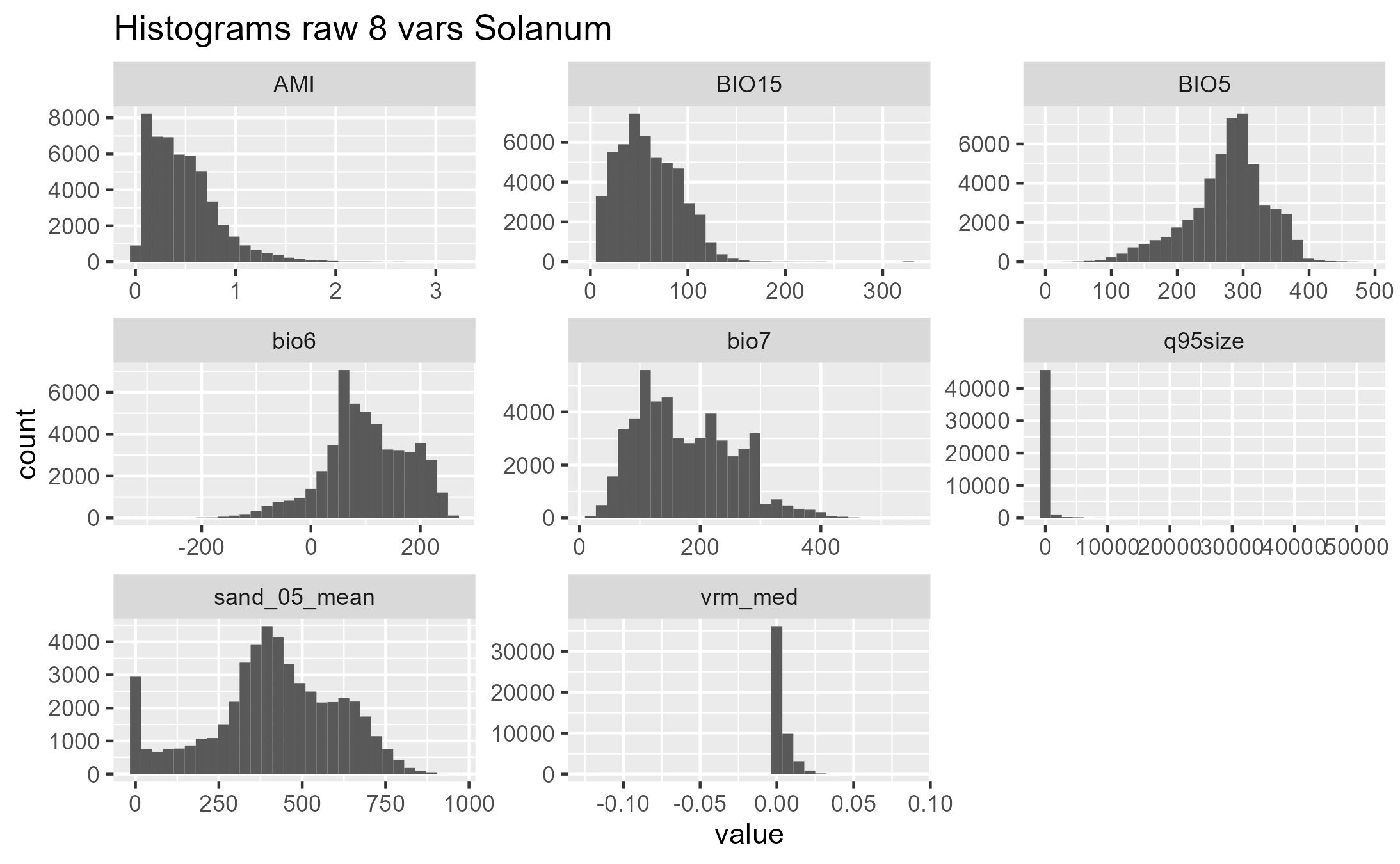

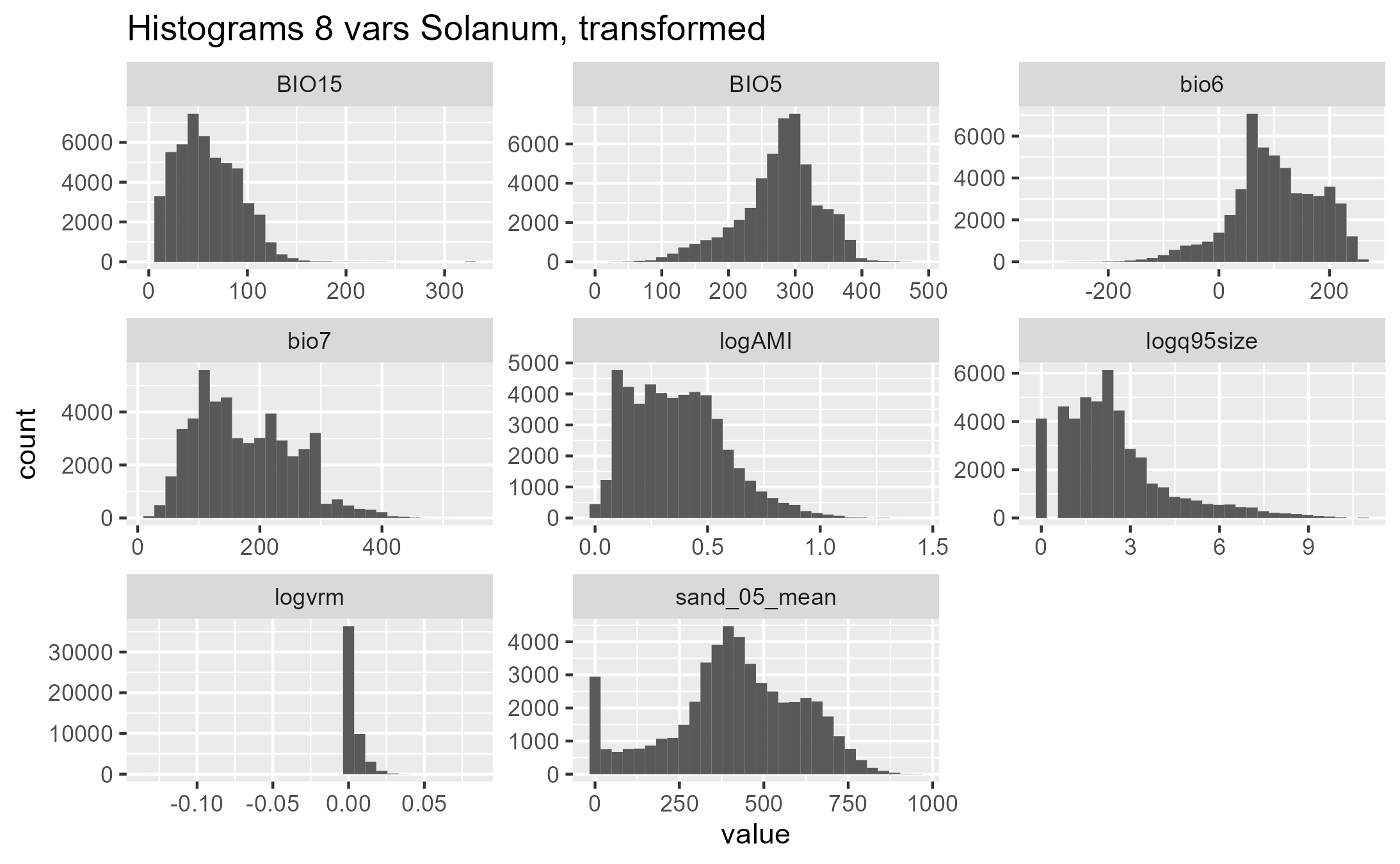


## Supplementary Figure 2. (a) Circle of contribution for PCA analysis; (b) Percentage of explained variance for each principal component in the PCA analysis.


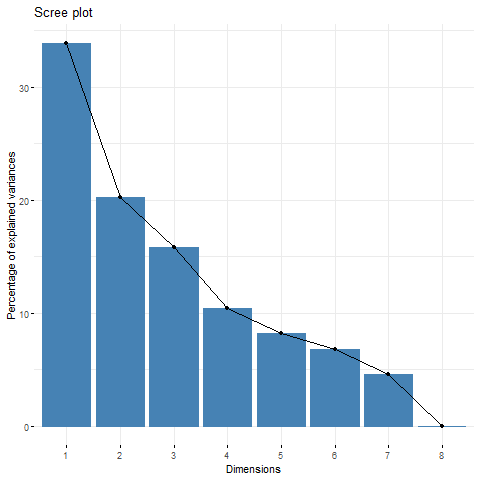

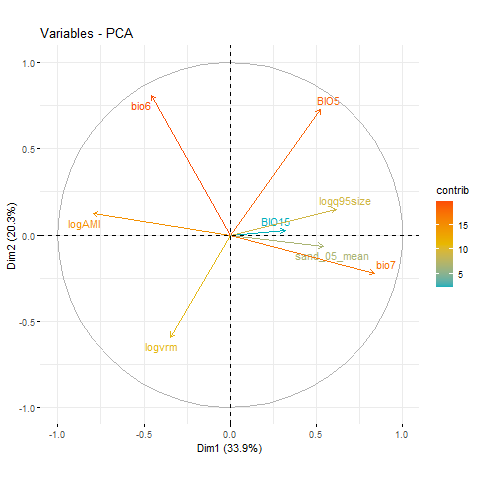


## Supplementary Figure S3. Boxplots of log-transformed niche breadth values (combined + individual variables) and range sizes, according to three growth forms.


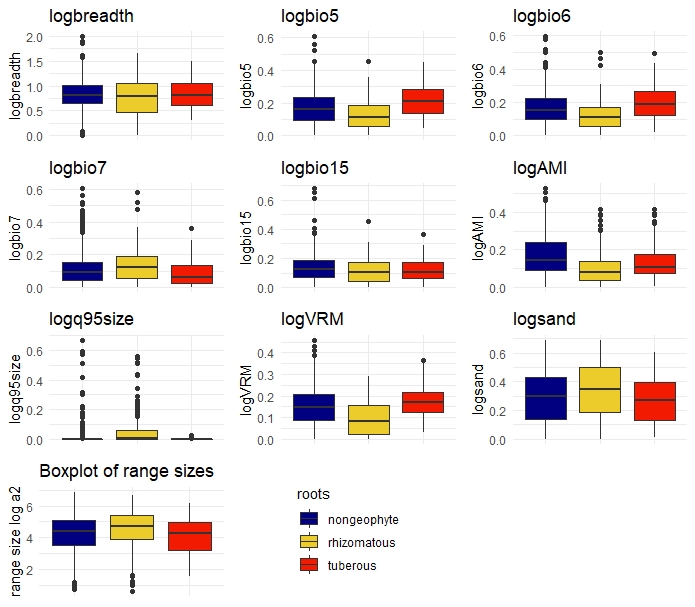

Supplement: Supplementary file 3 [file DataSheet1.docx]
